# Falciform ligament abscess from left sided portal pyaemia following malignant obstructive cholangitis

**DOI:** 10.1186/1477-7819-10-278

**Published:** 2012-12-22

**Authors:** Leigh R Warren, Manju D Chandrasegaram, Daniel J Madigan, Paul M Dolan, Eu L Neo, Christopher S Worthley

**Affiliations:** 1 Hepatobiliary Unit, Royal Adelaide Hospital, North Terrace, Adelaide, 5000, South Australia; 2 Department of Radiology, Royal Adelaide Hospital, North Terrace, Adelaide, 5000, South Australia

**Keywords:** Falciform ligament abscess, Round ligament abscess, Cholangitis, Ampullary carcinoma, Pancreatic carcinoma

## Abstract

Abscess formation of the falciform ligament is incredibly rare and perplexing when encountered for the first time. It is reported to occur in the setting of cholecystitis and cholangitis, but the pathophysiology is poorly understood.

In this case report, we present a 73-year-old man with falciform ligament abscess following cholangitis from an obstructive ampullary carcinoma. The patient was referred to the Royal Adelaide Hospital from a country hospital, with progressive jaundice, anorexia and nausea. Prior to transfer, he deteriorated with cholangitis, dehydration and renal failure. On arrival, his abdomen was exquisitely tender along the course of the falciform ligament. His blood tests revealed an elevated white cell count of 14.9 x 10^3^/μl, bilirubin of 291μmol/l and creatinine of 347 μmol/l. His CA 19-9 was markedly elevated at 35,000 kU/l. A non-contrast computed tomography (CT) demonstrated gross biliary dilatation and a collection tracking along the path of the falciform ligament to the umbilicus.

The patient was commenced on intravenous antibiotics and underwent an urgent endoscopic retrograde cholangiopancreatogram (ERCP) with sphincterotomy and biliary stent drainage. Cholangiogram revealed a grossly dilated biliary tree, with abrupt transition at the ampulla, which on biopsy confirmed an obstructing ampullary carcinoma. Following ERCP, his jaundice and abdominal tenderness resolved. He was optimized over 4 weeks for an elective pancreaticoduodenectomy.

At operation, we found abscess transformation of the falciform ligament. Copious amounts of pus and necrotic material was drained. Part of the round ligament was resected along the undersurface of the liver. Histology showed that there was prominent histiocytic inflammation with granular acellular eosinophilic components. The patient recovered slowly but uneventfully.

A contrast CT scan undertaken 2 weeks post-operatively (approximately 7 weeks after the initial CT) revealed left portal venous thrombosis, which was likely to be a delayed discovery and was managed conservatively.

We present this patient’s operative images and radiographic findings, which may explain the pathophysiology behind this rare complication. We hypothesize that cholangitis, with secondary portal pyaemia and tracking via the paraumbilical veins, can cause infectious seeding of the falciform ligament, with consequent abscess formation.

## Background

Abscess formation of the falciform ligament is incredibly rare and perplexing when encountered for the first time. It has been reported to occur in the setting of cholecystitis and cholangitis, but the pathophysiology of this occurrence is poorly understood. In this case report, we present a 73-year-old man with falciform ligament abscess following cholangitis from an obstructive ampullary carcinoma, whose clinical presentation we believe sheds light on the pathophysiology of falciform abscess formation.

## Case report

### Clinical history and examination

A 73-year-old man was referred to the Royal Adelaide Hospital from a country hospital, with a 2-week history of progressive jaundice, anorexia and nausea. Prior to transfer, the patient deteriorated with worsening jaundice, cholangitis, tachycardia, dehydration and renal failure. His comorbidites included morbid obesity, hypertension and a blind left eye from previous trauma. On arrival, his abdomen was exquisitely tender to palpation and percussion along the upper midline, with tenderness along the liver margin.

### Blood tests

The patient’s blood tests demonstrated an elevated white cell count of 14.9 x 10^3^/μl, bilirubin of 291μmol/l and creatinine of 347 μmol/l. His CA 19-9 was markedly elevated at 35,000 kU/l. 

### Imaging

**Figure 1:**
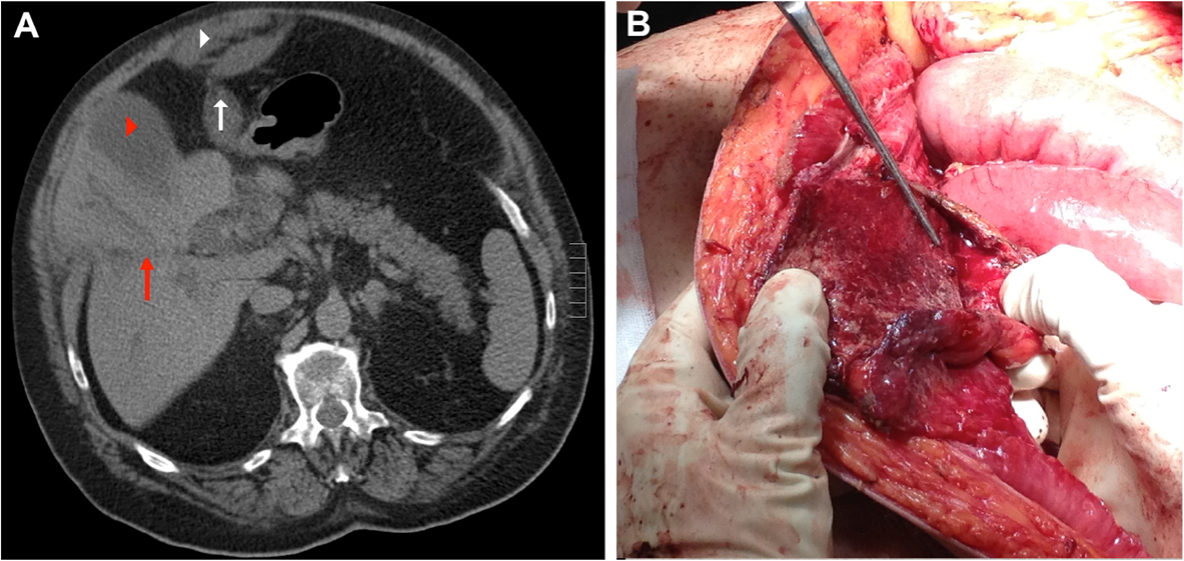
**CT abdomen. **(**A**) The enlarged gall bladder (red arrow head) and dilatation of intrahepatic bile ducts (red arrow). A fluid collection is seen tracking from the liver along the path of the falciform ligament (white arrow) to a collection on the anterior abdomen (white arrow head). (**B**) Operative view of the rind of thickened inflammatory falciform tissue (black arrow) after abscess drainage.

A non-contrast computed tomography (CT) was performed due to renal failure. This demonstrated gross biliary dilatation and a collection that appeared to track along the path of the falciform ligament to the umbilicus (Figure 1A).

### Endoscopic diagnosis and intervention

The patient was commenced on intravenous antibiotics, and underwent an urgent endoscopic retrograde cholangiopancreatogram (ERCP) with sphincterotomy and biliary stent drainage. Cholangiogram revealed a grossly dilated biliary tree, with abrupt transition at the ampulla. There was no evidence of contrast leakage from the intrahepatic biliary radicles. Biopsy confirmed an obstructing ampullary carcinoma. He recovered well following ERCP, and his jaundice and abdominal tenderness settled. He was treated with antibiotics and was optimized over 4 weeks for an elective pancreaticoduodenectomy.

### Pancreaticoduodenectomy and drainage of falciform abscess

**Figure 2:**
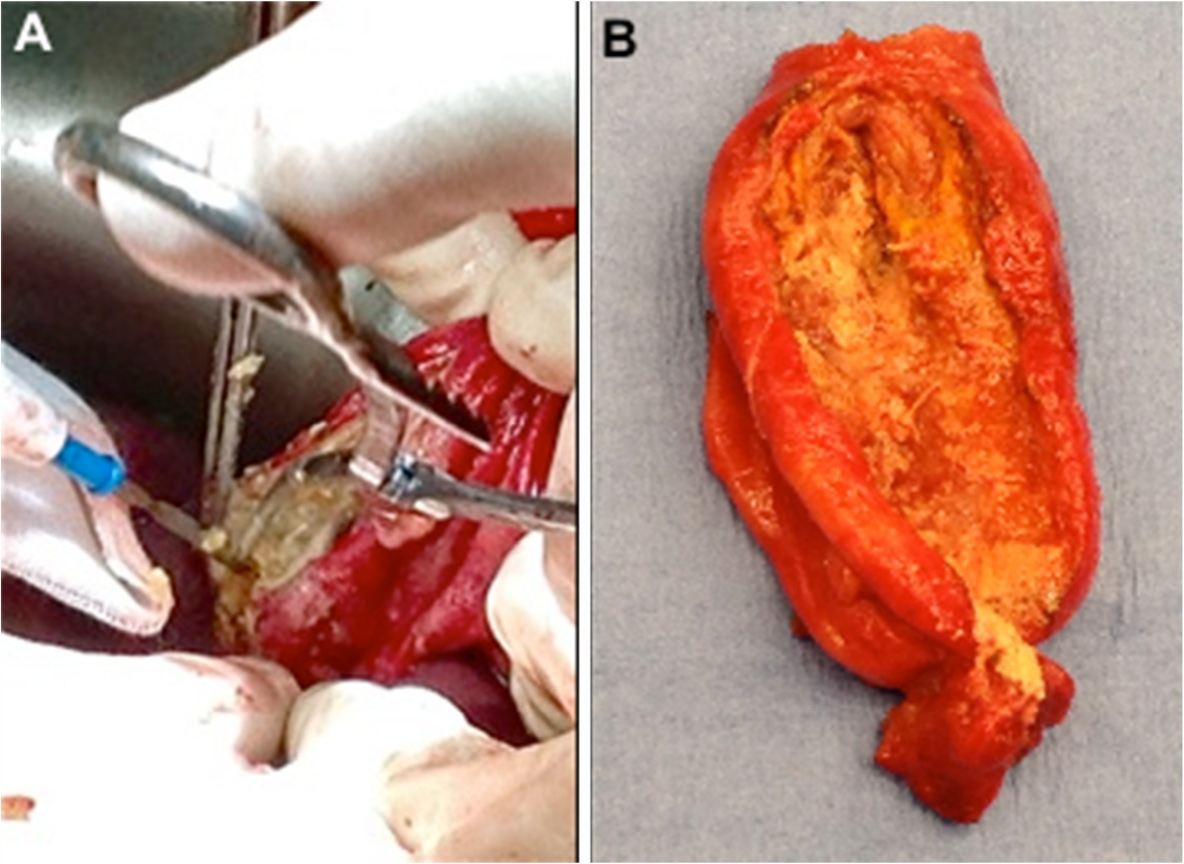
**Operative view. **(**A**) Necrotic tissue excised from the round ligament. (**B**) The eventually resected round ligament.

At operation, we found abscess transformation of the falciform ligament. Copious amounts of pus and necrotic material was drained (Figure 2). The enveloping layers of the falciform ligament had formed a thick rind extending from the umbilicus to the liver (Figure 1A). Part of the round ligament was resected along the undersurface of the liver (Figure 2B). Histology showed that there was prominent histiocytic inflammation with granular acellular eosinophilic components. No organisms grew on culture.

### Recovery

**Figure 3:**
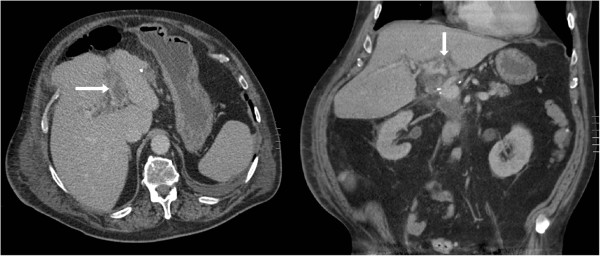
Postoperative CT abdomen with contrast showing left portal venous thrombosis (arrows) on axial and sagittal views.

A contrast CT scan was performed at 2 weeks following pancreaticoduodenectomy (approximately 7 weeks after the initial non-contrast CT scan). This demonstrated resolution of his collection and evidence of left sided portal thrombosis, which extended into the left portal branches (Figure 3). The left sided portal thrombosis was likely to have been a delayed discovery and was managed conservatively without anticoagulation. The patient recovered slowly but uneventfully and remained well at 8 months follow-up.

## Discussion

Pathologic lesions of the falciform ligament were first described in 1909 [1] and there have been few reports in the literature since. Falciform ligament abscess presents a difficult and perplexing problem when encountered clinically for the first time. It has been reported to occur in the setting of cholecystitis [2,3], cholecystolithiasis [4,5] and cholangitis [6]. The pathophysiology of this occurring secondary to cholecystitis or cholangitis is poorly understood [1].

Biliary infection or thrombophlebitis may spread via the portal venous system. In general, the cholecystic veins flow directly into the portal vein and the pericholedochal venous system [7,8]. We suspect that this patient may have had left sided portal pyaemia secondary to cholangitis, with spread of this infection via the paraumbilical veins to the falciform ligament, resulting in abscess formation.

The paraumbilical vein is most relevant in portal hypertension, when recanalization of the paraumbilical vein enables spontaneous portosystemic collateralization, known as Cruveilhier-Baumgarten syndrome [9,10]. The paraumbilical vein originates from the umbilical portion of the left portal vein in the falciform ligament, and heads towards the umbilicus and periumbilical veins [11].

This patient’s left sided portal vein thrombosis may have been a consequence of thrombophlebitis from cholangitis [12]. This was managed without anticoagulation since it was likely to have been a delayed finding from his initial septic process, 7 weeks prior. The thrombosis extended into the portal branches of the left portal vein, and the patient was asymptomatic with no clinical consequence from this, both at time of discovery and after.

The abscess in this patient may have been sterilized following his initial treatment with biliary drainage and intravenous antibiotics, which would explain why culture and histological assessment did not reveal bacterial contamination.

## Conclusions

Falciform ligament abscess should be considered as a rare but important complication of biliary tract obstruction and infection. In this setting, it may present with upper midline abdominal pain. Diagnosis is largely made on imaging and treatment may be surgical, although improvement with endoscopic biliary drainage and antibiotics was effective in this case.

## Consent

Written informed consent was obtained from the patient for publication of this report and any accompanying images.

## Abbreviations

CT: Computed tomography; ERCP: Endoscopic retrograde cholangiopancreatogram.

## Competing interest

The authors declare that they have no competing interest.

## Authors’ contributions

All authors were involved in the care of the patient and reviewed and edited the manuscript. All authors read and approved the final manuscript.

## Presentation

This case has been presented as a poster in the Australian Health and Medical Research Congress 2012.
